# Chitosan microparticle-based antibacterial nasal barrier formulation and in vivo evaluations

**DOI:** 10.55730/1300-0527.3908

**Published:** 2026-06-03

**Authors:** Güliz AK, Ayça EREK, Tuğba KARAKAYALI, Şadiye ÇETİN, Şenay ŞANLIER

**Affiliations:** 1 Department of Biochemistry, Faculty of Science, Ege University, İzmir, Turkiye; 2 Department of Pharmaceutical Biotechnology, Faculty of Pharmacy, Trakya University, Edirne, Turkiye; 3 Department of Biochemistry, Graduate School of Natural and Applied Sciences, Ege University, İzmir, Turkiye; 4 Santek Medical Products, Consultant, Software, Hardware, and Computer Services Industry and Trade Ltd. Co., İzmir, Turkiye

**Keywords:** Chitosan microparticles, nasal barrier formulation, olive leaf extract, thyme oil, spray drying, antibacterial activity

## Abstract

Microorganisms remain a major cause of infectious diseases, emphasizing the need for alternative and complementary antimicrobial strategies. In this context, plant-derived materials have attracted increasing attention for their antimicrobial and protective properties. Chitosan, a biocompatible and cationic biopolymer, has emerged as an effective carrier system for encapsulating such plant-derived components. In this study, chitosan microparticles were prepared through ionic gelation followed by spray drying and loaded with thyme oil and olive leaf extract. The resulting microparticles exhibited an average size of approximately 1.8 μm and a total phenolic content of 23 mg GAE/g. These microparticles were incorporated into a glycerin–starch ointment to develop a nasal barrier formulation. The formulation showed physicochemical properties compatible with intranasal administration, with pH values in the range of 5–7. The antibacterial activity of the formulation was tested in vivo using BALB/c mice, in which a time-dependent reduction in microbial viability was observed after intranasal administration. Overall, these results suggest that chitosan-based microparticle systems containing plant-derived components could serve as nasal barrier formulations with supportive antibacterial effects.

## 1. Introduction

Microorganisms are a major cause of infectious diseases worldwide. Throughout history, infectious diseases have posed a persistent threat to human health worldwide. These diseases contribute to substantial mortality as well as social and economic burdens [1]. The increasing incidence of emerging infectious diseases and pandemic events has highlighted the need to identify new antimicrobial and antiviral compounds, as well as to develop effective antimicrobial materials [2]. Increasing bacterial resistance to antibiotics due to the extensive use of antibiotics and other chemically synthesized antimicrobials has revived interest in plant products as alternative antimicrobial agents to control pathogenic microorganisms [3]. Essential oils are aromatic and volatile secondary metabolites of plants that have been widely investigated for their natural antimicrobial properties [4]. Among the various essential oils, thyme essential oil, obtained from *Origanum vulgare* L., is well known for its antimicrobial and antioxidant activities. These biological activities are mainly attributed to phenolic compounds such as carvacrol and thymol, which are the major constituents of thyme essential oil, as well as to monoterpene hydrocarbons including p-cymene and γ-terpinene present at lower concentrations [5,6]. Olive leaves are a widely available and cost-effective natural source of phenolic compounds. The phenolic compounds present in olive leaves exhibit antimicrobial activity. Olive leaves also contain bioactive compounds such as secoiridoids (e.g., oleuropein) and flavonoids. Flavonoids have been reported to inhibit or inactivate various bacterial strains, interfere with key viral enzymes such as reverse transcriptase and protease, and exhibit activity against certain pathogenic protozoa [7]. Despite their widespread use, essential oils present several limitations in direct applications, including rapid loss through evaporation and reduced stability upon exposure to ultraviolet light and oxygen. These limitations can be mitigated by surrounding essential oils with a polymeric matrix through microencapsulation or microspherization techniques [8]. Chitosan is a naturally derived biopolymer obtained through the N-deacetylation of chitin, a β-(1–4)-linked polymer of N-acetyl-D-glucosamine. Its nontoxic, biodegradable, and biocompatible nature makes chitosan an attractive candidate for use as a polymeric carrier in microencapsulation systems for various biotechnological applications.

Cationic polymers have been reported to offer several advantages over small-molecule antibiotics, including sustained inhibitory effects, broad-spectrum antibacterial activity, and improved biocompatibility. Accordingly, the cationic character of chitosan, arising from its reactive functional groups, contributes to its potential antibacterial activity [9,10]. The COVID-19 pandemic has accelerated efforts to develop nasal products that can contribute to limiting the spread of infectious agents. In this context, increasing attention has been directed toward intranasal barrier formulations, as the nasal cavity represents a primary entry site for airborne pathogens. Various nasal barrier products have been developed to reduce or prevent pathogen access to the nasal epithelium. Unlike conventional antimicrobial agents, the primary aim of these formulations is not to exert a direct and specific effect on the pathogen; instead, they are designed to form a physical barrier on the nasal mucosa, limiting pathogen–host interactions at the epithelial surface. Such barrier-based approaches have therefore emerged as a promising and complementary strategy for reducing microbial colonization in the upper respiratory tract [11–13]. Despite the increasing interest in plant-derived antimicrobial agents and chitosan-based carrier systems, most studies have focused on either the antimicrobial activity of natural compounds or their incorporation into delivery systems separately. Similarly, nasal formulations have generally been developed either as drug delivery systems or as barrier-forming products, with limited integration of both approaches into a single formulation.

In the present study, a nasal barrier formulation was developed based on chitosan microparticles (CMs) prepared by spray drying and loaded with plant-derived compounds, i.e. olive leaf extract and thyme oil. The novelty of our work lies in the integration of a microparticle-based delivery system containing plant-derived bioactive compounds with a barrier-forming semisolid nasal formulation, enabling both physical barrier formation on the nasal mucosa and supportive antibacterial activity. The microparticle-based ointment developed was characterized in terms of its physicochemical properties and evaluated for its in vivo antibacterial efficacy and suitability for intranasal application.

## 2. Materials and methods

### 2.1. Materials

Acetic acid was purchased from Tekkim. Chitosan (degree of deacetylation >85%) and sodium hydroxide were obtained from Sigma-Aldrich. Ethanol, methanol, Folin–Ciocalteu reagent, sodium carbonate, and gallic acid were purchased from Merck. Olive leaves were obtained from Arifoğlu and thyme oil was supplied by Santek Medikal.

### 2.2. Preparation of olive leaf extract

The olive leaves were ground into a fine powder and homogenized at 8000 rpm for 1 min using an Ultra-Turrax T-10S homogenizer with 25 mL of 80% (v/v) methanol. The resulting homogenate was placed in an orbital shaker and stirred at 720 rpm for 30 min. After centrifugation at 3000 rpm for 10 min, the supernatant was collected and filtered through a 0.2-μm PTFE membrane filter. The olive leaf extract (OLE) obtained was stored at 4 °C until further use.

### 2.3. Preparation of spray-dried CMs

A 0.2% (w/v) chitosan solution was prepared by dissolving chitosan in 1% (v/v) acetic acid with continuous stirring. The pH was adjusted to 4.6–4.8 using sodium hydroxide. To mimic the solvent conditions used during encapsulation, 5 mL of ethanol and 2 mL of distilled water were added to 19 mL of the chitosan solution. The mixture was stirred for 1 h and then spray-dried in a Büchi Mini Spray Dryer B-290 (Büchi, Germany) using air as the drying medium.

The spray-drying conditions were set as follows: inlet temperature of 160 °C, aspiration rate of 90%, feed rate of 10%, and solution flow rate of 50–60. The outlet temperature ranged from 82 to 85 °C [14]. These processing parameters were chosen based on preliminary experiments. The resulting CMs were collected as a fine powder and stored in a desiccator until further analysis.

### 2.4. Preparation of olive leaf extract and thyme oil encapsulated CMs

To prepare olive leaf extract and thyme essential oil encapsulated in CMs (OTC), 19 mg of olive leaf extract was dissolved in 2 mL of distilled water and 4 mL of ethanol. Separately, 19 mg of thyme essential oil was dissolved in 1 mL of ethanol. The olive leaf extract and thyme oil solutions were combined and thoroughly mixed. Afterwards, the resulting mixture was added to 19 mL of the chitosan solution with continuous stirring [15,16].

The formulation was stirred for 1 h at room temperature to ensure even distribution of the encapsulated compounds within the chitosan solution and then spray-dried using a Büchi Mini Spray Dryer B-290 (Büchi, Germany) under the same conditions used for preparing blank CMs. The resulting OTC microparticles were collected as a fine powder and stored in a desiccator until further analysis.

### 2.5. Characterization of OTC microparticles

The total phenolic content of the OTC microparticles was measured spectrophotometrically using the Folin–Ciocalteu method, as previously described [15,17]. Briefly, the samples were reacted with the Folin–Ciocalteu reagent and absorbance was recorded at 765 nm with a UV–Vis spectrophotometer (Agilent Technologies, Cary 60 UV–Vis).

Total phenolic content was measured using a gallic acid calibration curve and expressed as milligrams of gallic acid equivalents per gram of product (mg GAE/g). The calibration curve equation was y = 8757x, with an R^2^ value of 0.9995. Encapsulation efficiency was determined based on total phenolic content using Equation (1), which expresses the ratio of phenolic compounds retained within the microparticles to the total phenolic compounds initially present in the formulation mixture before spray drying.


(1) $$\text{Encapsulation efficiency }(\%)=\frac{\text{Phenolic compound amount in microparticles}}{\text{total phenolic compound in formulation mixture}}\times 100$$

Particle size distributions of the blank CMs and OTC microparticles were measured using a laser diffraction particle size analyzer (Malvern Mastersizer Hydro 3000). Measurements were conducted with distilled water as the dispersion medium.

The surface morphology of the CMs and OTC microparticles was examined using scanning electron microscopy (SEM). Before imaging, samples were mounted on aluminum stubs and sputter-coated with gold. The SEM analysis was conducted with a Thermo Scientific Apreo S microscope.

Fourier transform infrared (FT-IR) spectroscopy was used to examine the chemical structures of the CMs and OTC microparticles and to evaluate potential interactions between chitosan and the encapsulated compounds. FT-IR spectra were obtained with a PerkinElmer Spectrum Two spectrophotometer in the range of 400–4000 cm^−1^.

### 2.6. Nasal formulation of olive leaf extract and thyme oil encapsulated CMs

#### 2.6.1. Preparation of ointment

A starch-based ointment was prepared. Briefly, 10 g of starch was dispersed in a small volume of cold distilled water to obtain a uniform suspension. The remaining amount of distilled water (total water content: 70 g) was heated to boiling, and the starch suspension was added to the hot water under continuous stirring to induce gelatinization.

The resulting mixture was allowed to cool to a warm temperature, after which 20 g of glycerin was added and mixed thoroughly until a homogeneous ointment base was obtained. The formulation was continuously stirred during cooling to room temperature, allowing the ointment to develop its final consistency gradually.

Upon cooling, the ointment base prepared was transferred into a sealed container and stored at 25 °C until further use.

#### 2.6.2. Formulation of an ointment containing OTC microparticles

OTC microparticle-containing ointment formulations were prepared at various concentrations to identify a suitable formulation for intranasal application. OTC microparticles were incorporated into the starch-based ointment base at concentrations of 0.25%, 0.5%, 1%, and 2% (w/w).

For each formulation, the calculated amount of OTC microparticles was added to the ointment base followed by mixing with a magnetic stirrer at room temperature until the microparticles were evenly dispersed. All ointment formulations were prepared in a biosafety cabinet to minimize the risk of microbial contamination prior to in vivo application.

All formulated OTC microparticle-containing ointments were evaluated for viscosity and pH. Viscosity measurements were performed at room temperature using a Brookfield DV-II+ Pro viscometer equipped with a TD spindle at 200 rpm. The viscosity data were recorded in centipoises (cP). The pH values of the formulations were measured using a pH meter. Based on the viscosity and pH results, one formulation was selected for subsequent in vivo studies.

#### 2.6.3. In vivo antibacterial efficacy evaluation of the ointment formulations

All experimental procedures were approved by the Ege University Animal Experiments Local Ethics Committee (Ethics Committee Approval No. 2020-123).

A total of 15 male and female BALB/c mice (6–8 weeks old) were obtained from Kobay Experimental Animals Laboratory Inc. The animals were housed under controlled environmental conditions (temperature 24 ± 1 °C, relative humidity 50 ± 10%) with a 12-h light/12-h dark cycle. The mice were randomly assigned to five experimental groups (n = 3 per group).

To establish nasal bacterial colonization, each mouse was inoculated intranasally with *Serratia marcescens*. A suspension containing 1 × 10^6^ CFU in 10 μL was administered into the same nostril using a pipette. This inoculation procedure was performed three times at 10-min intervals [18]. Ten minutes after the final inoculation, 20 mg of the respective ointment formulation was administered intranasally using a pipette.

All test formulations were prepared in the starch-based ointment base. The experimental groups were defined as follows:

Group 1 (OTC ointment): olive leaf extract and thyme oil encapsulated CMs incorporated into the ointment base
Group 2 (OLE ointment): olive leaf extract encapsulated CMs incorporated into the ointment base
Group 3 (Thyme ointment): thyme oil encapsulated CMs incorporated into the ointment base
Group 4 (Chitosan ointment): blank CMs incorporated into the ointment base
Group 5 (Control ointment): phosphate-buffered saline (PBS) incorporated into the ointment base

The formulation composition and abbreviations are summarized in Table 1.

Before administration of the ointment formulations, a nasal swab sample was collected from each mouse to determine the baseline bacterial load (t = 0). Following treatment, additional nasal swab samples were collected at 5, 15, and 30 min postadministration to evaluate short-term antibacterial effects.

Collected swab samples were microbiologically analyzed using the pour plate method, as described by Sanders [19]. Bacterial enumeration was performed at the EGEMIKAL Analysis Laboratory, Faculty of Science, Ege University, Türkiye. Bacterial viability was expressed as a percentage by normalizing colony counts obtained at each time point to the baseline value (t = 0), which was set at 100%.

## 3. Results and discussion

### 3.1. Characterization of OTC microparticles

#### 3.1.1. Total phenolic compounds in CMs

The phenolic compounds encapsulated within the OTC microparticles were quantified after acid hydrolysis of the chitosan matrix, followed by analysis using the Folin–Ciocalteu method. Based on the interaction of the released phenolic compounds with the Folin–Ciocalteu reagent, the total phenolic content of the final spray-dried OTC product was determined as 23 mg/g GAE. In addition, the loading efficiency of phenolic compounds, expressed in terms of GAE, was calculated as 22.90%. The loading efficiency obtained is consistent with previously reported values for OTC microparticles prepared by spray drying, which were reported to be approximately 27% (GAE) by Kosaraju et al. [20].

#### 3.1.2. FTIR

**Figure 1:**
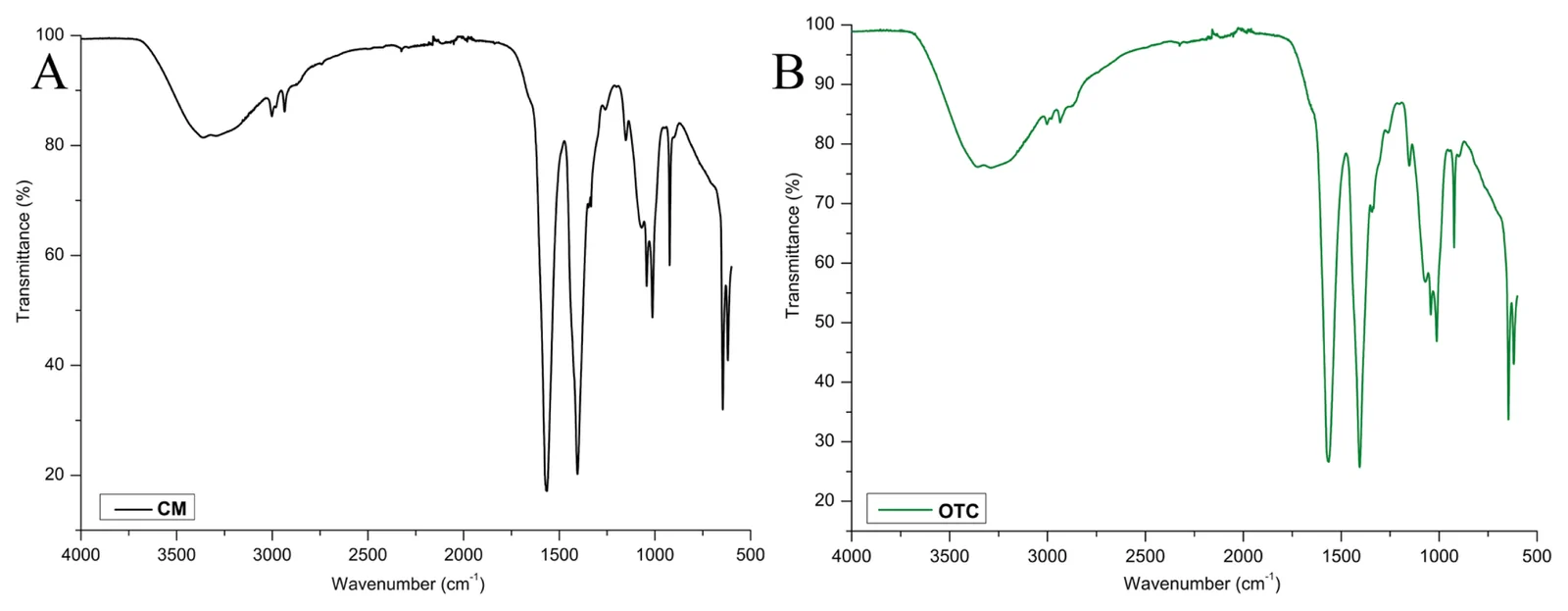
FT-IR spectra of CMs (A) and OTC microparticles (B).

FT-IR spectroscopy was employed to evaluate the chemical structure of CMs and OTC microparticles. Figure 1A presents the FT-IR spectrum of the CMs, while Figure 1B shows the FT-IR spectrum of the OTC microparticles.

The FT-IR spectrum of the CMs exhibited a broad absorption band in the range of 3500–3200 cm^−1^, which can be attributed to the overlapping stretching vibrations of hydroxyl O–H and amino (N–H) groups characteristic of chitosan. Bands observed around 2900 cm^−1^ correspond to C–H stretching vibrations of the polysaccharide backbone. In addition, characteristic absorption bands detected in the 1650–1550 cm^−1^ region are associated with amide vibrations originating from the chitosan structure [21].

The FT-IR spectrum of the OTC microparticles showed a profile similar to that of the CMs, indicating that the spray-drying process did not alter the chitosan matrix’s fundamental chemical structure. However, noticeable changes in band intensity and slight shifts were observed, particularly in the 3500–3000 cm^−1^ region, compared to the CMs. These variations may be attributed to the presence of encapsulated components within the CMs and their influence on the local environment of functional groups [22]. Furthermore, differences between the spectra of the CMs and OTC microparticles were also observed in the fingerprint region (1200–800 cm^−1^). Such spectral variations are commonly associated with the incorporation of additional components into polymeric matrices and indicate modifications in the vibrational environment without chemical degradation of the carrier structure [23]. Overall, the FT-IR results demonstrate the structural integrity of the chitosan matrix after spray drying and support the successful incorporation of olive leaf extract and thyme oil into the CMs, as evidenced by the observed spectral changes and the compatibility between the components.

#### 3.1.3. Microparticle size and morphology analysis

**Figure 2:**
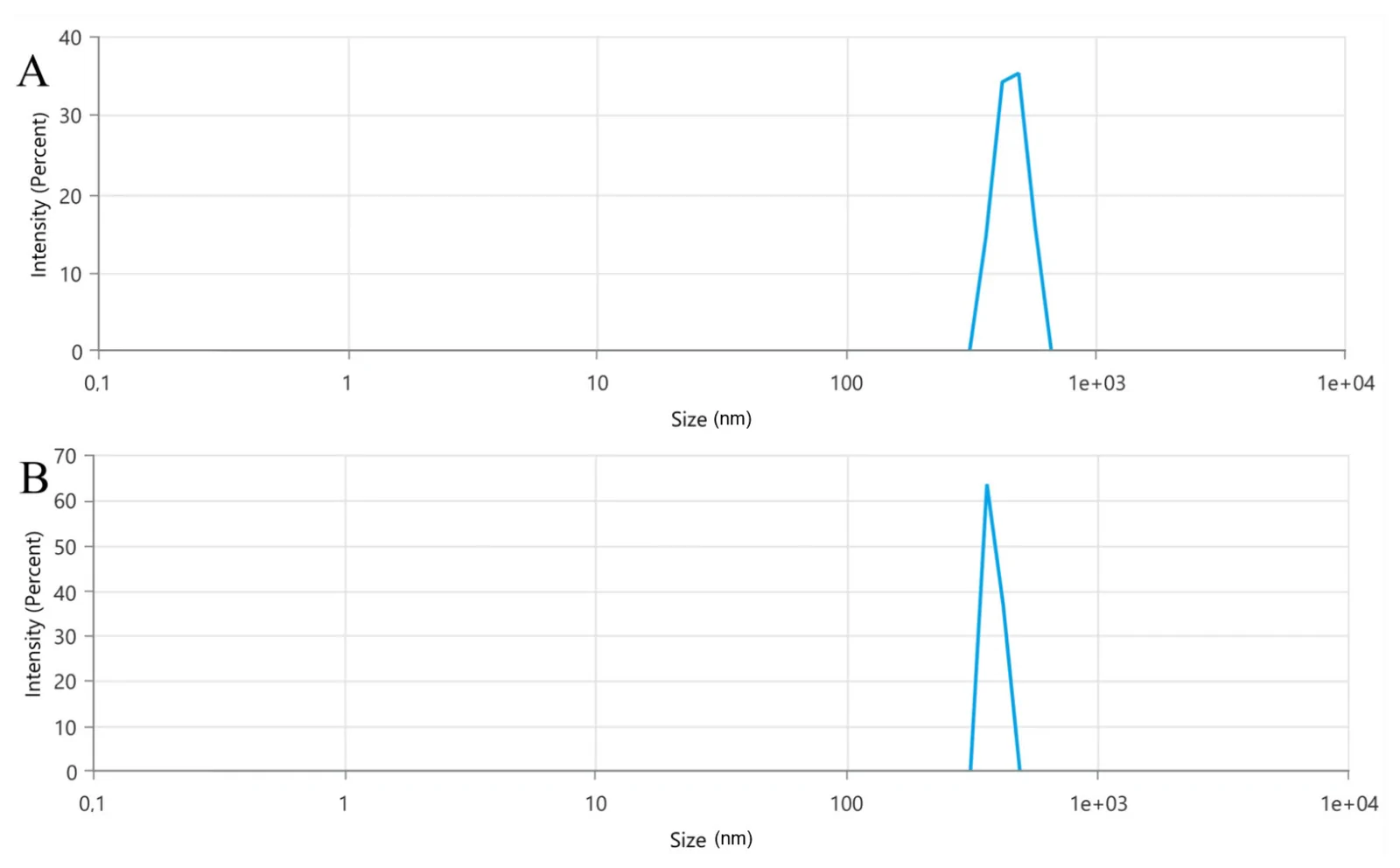
Hydrodynamic size distribution graphs of CMs (A) and OTC microparticles (B).

The hydrodynamic size distributions of the CMs and OTC microparticles are shown in Figures 2A and 2B, respectively. According to hydrodynamic size analysis, the average particle size of the CMs was 879.23 ± 86 nm with a PDI value of 0.63 ± 0.04, whereas the average hydrodynamic size of the OTC microparticles was 1.867 ± 0.25 μm with a PDI value of 0.80 ± 0.03.

The increase in the particle size of the OTC microparticles compared to the CMs may be associated with the incorporation of olive leaf extract and thyme oil.

**Figure 3:**
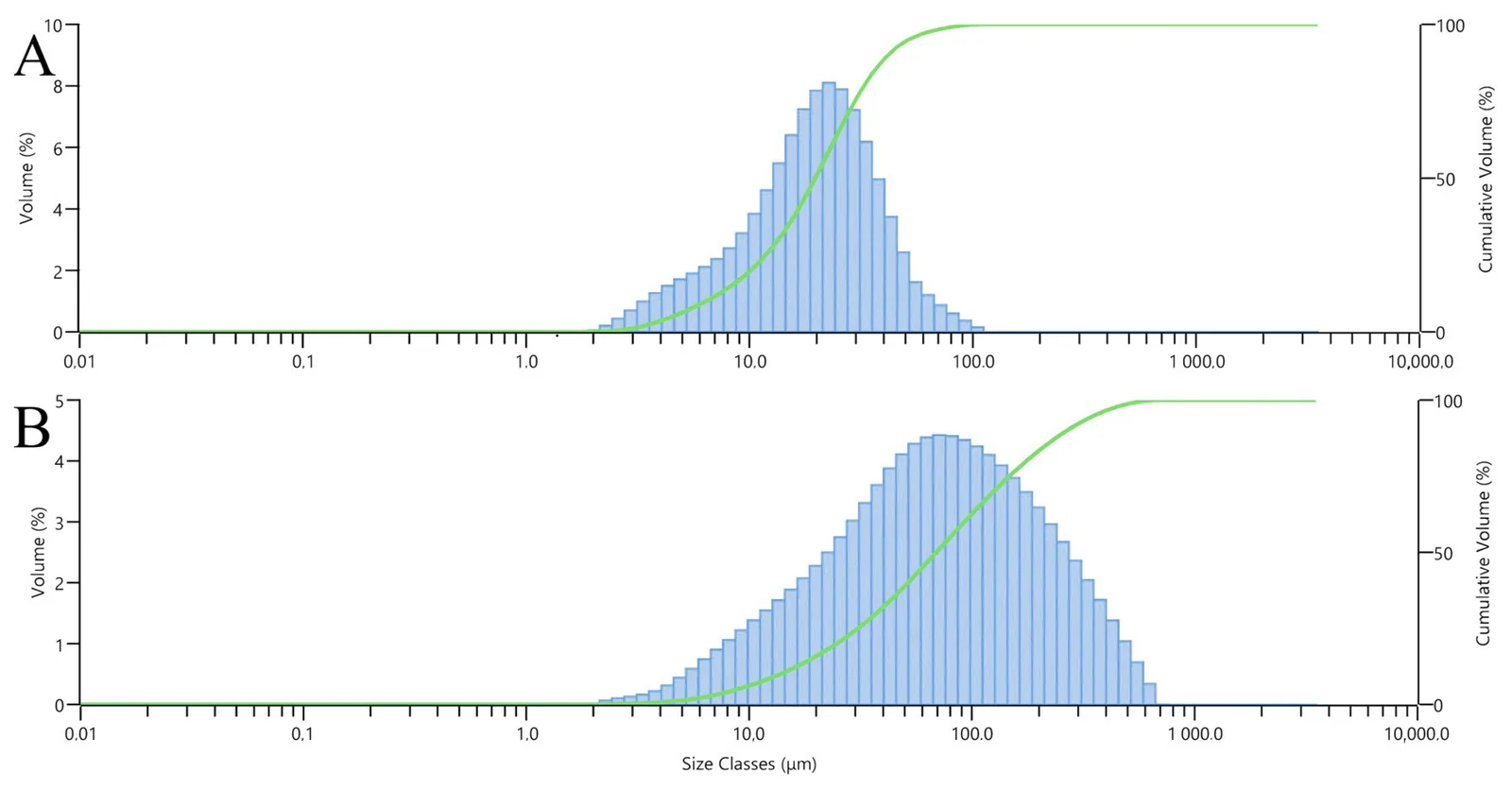
Particle size distribution profiles of (A) CMs and (B) OTC microparticles obtained by mastersizer analysis.

Particle size distribution analyses of the CMs and OTC microparticles prepared were performed using a Mastersizer particle size analyzer. The particle size distribution profile of the CMs is presented in Figure 3A. As shown in the graph, 94.46% of the microparticles were within the size range of 0.1–50 μm, while approximately 30% were distributed between 2.13 and 12.7 μm. The particle size distribution profile of the OTC microparticles is shown in Figure 3B. According to the results, 38.99% of the microparticles were in the range of 0.1–50 μm and approximately 10% were distributed between 2.42 and 12.7 μm.

**Figure 4:**
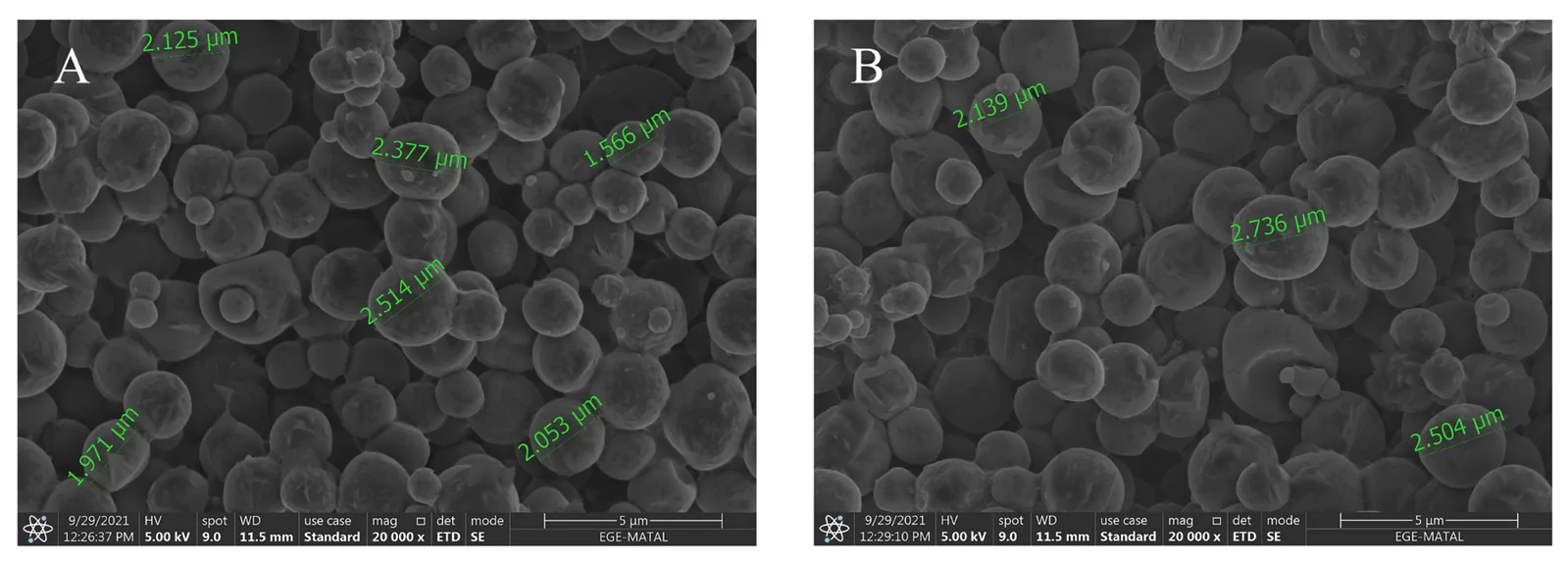
SEM images of (A) CMs and (B) OTC microparticles.

The morphology of the microparticles was evaluated using SEM. SEM images of spray-dried CMs, shown in Figure 4A, and OTC microparticles, shown in Figure 4B, revealed that the microparticles exhibited a generally spherical shape with varying degrees of surface roughness. In the literature, it has been reported that CMs have a spherical, rough surface [24,25].

In the study by Silva Garcia et al., extracts from lemongrass and geranium were encapsulated in CMs prepared by spray drying, and the anti-Candida activity of the carrier system was investigated. The size of the microparticles containing the plant extracts was in the range of 1–15 μm [26].

In another study, Alhalaweh et al. synthesized CMs using the spray-drying method for a nasal formulation. The CMs obtained were spherical and ranged in size from 2 to 10 μm. The spherical shape formed as the droplet dries in the hot air stream during spray drying is characteristic of amorphous spray-dried powders [27]. Previous studies have shown that particles larger than 10 μm in diameter will likely accumulate effectively in the nasal cavity. As a result, most particles larger than 0.5 μm are collected in the nasal cavity with high efficiency following inhalation [28]. Based on these findings, the particle size and morphology of the OTC microparticles prepared suggest their potential suitability for nasal application.

### 3.2. Nasal formulation of olive leaf extract and thyme oil encapsulated CMs

A starch-based glycerin ointment was prepared for nasal administration. The formulation is intended to be applied to the nasal cavity to form a physical barrier on the nasal mucosa.

The formulation’s pH may affect the passage of the active ingredient through the nasal mucosa. In addition, the formulation pH should be adjusted to physiological conditions to minimize the risk of nasal irritation. The surface pH of the nasal mucosa is between 5.5 and 6.7 [29]. The pH of the developed ointment formulation was measured in the range of 6.5–7, which is compatible with the physiological pH of the nasal cavity. Viscosity is another parameter to be considered in the formulation’s physicochemical properties. A high viscosity of the formulation may increase the contact between the active substance and the nasal mucosa and, thus, the residence time of the active substance. It should also be ensured that the structure is homogeneous and semisolid [30]. The stability evaluation in our study was limited to physicochemical parameters, while long-term shelf-life studies were not included within the scope of the present work. After the ointment formulation was developed, specific ratios of OTC microparticles (0.25%, 0.5%, 1%, and 2% (w/w)) were added, and stability-related parameters were evaluated to support the selection of an appropriate formulation. Within the scope of stability tests, pH, viscosity parameters, and phase separation in ointment formulations containing OTC microparticles were examined [30]. The results of the pH and viscosity measurements are given in Table 2. According to Table 2, the pH was around 4 when 2% OTC microparticles were added. This pH value is lower than the physiological pH range of the nasal cavity. In formulations containing 0.25% and 0.5% OTC microparticles, the pH was around 6 and suitable for nasal application. In the formulation containing 1% OTC microparticles, the pH was around 5–5.5, which is also compatible with nasal pH. In addition, no significant change was observed in viscosity measurements. Based on the parameters evaluated, no pronounced instability was observed in this formulation compared to the other OTC microparticle-containing formulations. No phase separation was observed in the formulations when their appearance was analyzed. Based on pH, viscosity, physical appearance, and homogeneity evaluations, the ointment formulation containing 1% OTC microparticles was considered compatible with nasal administration and selected for subsequent in vivo experiments.

### 3.3. In vivo antibacterial efficacy evaluation of ointment formulations

**Figure 5:**
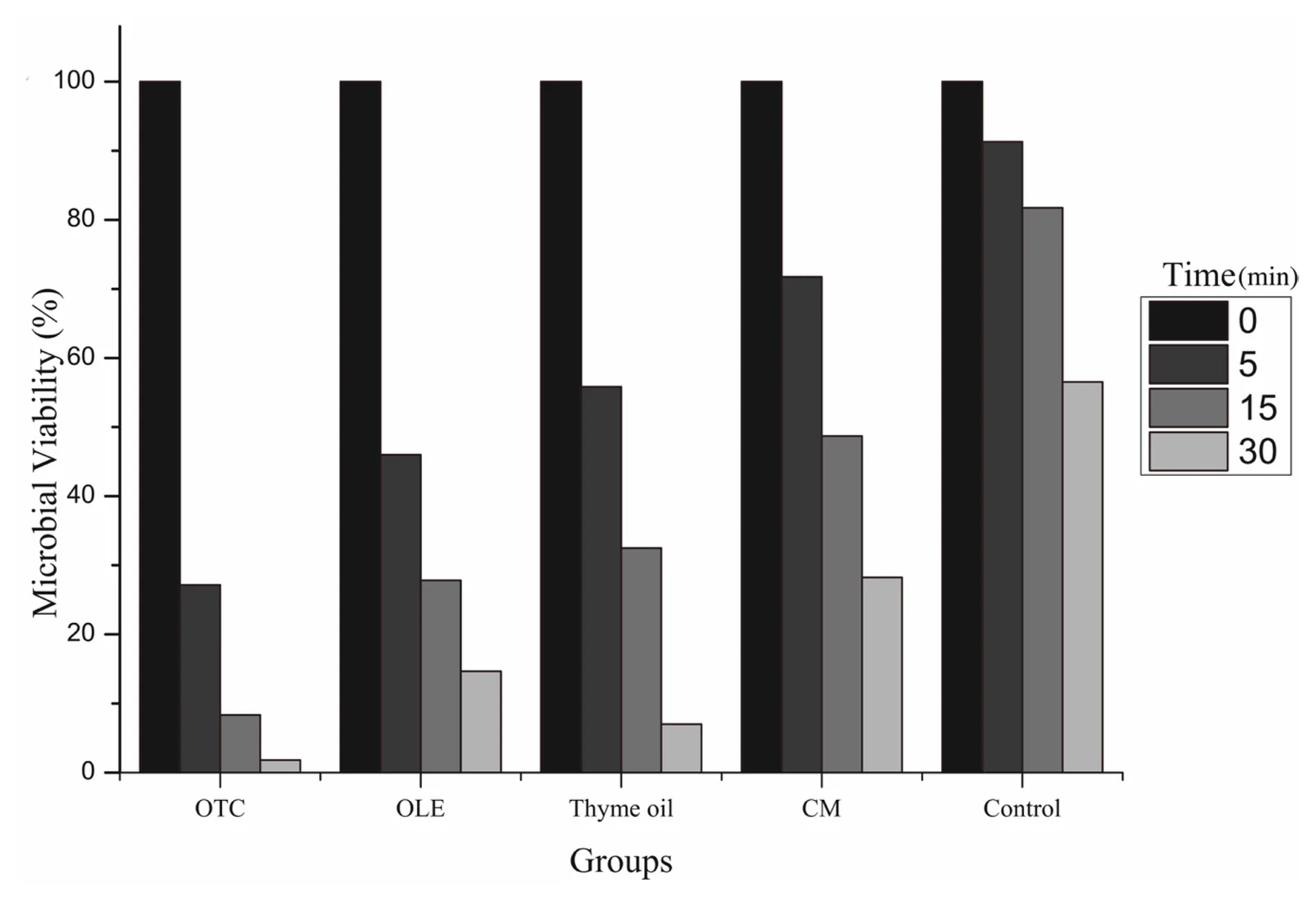
Effects of the formulations on microbial viability.

In vivo experiments were conducted to evaluate the antimicrobial efficacy of the formulations, and colony counts were obtained from nasal swab samples collected at defined time intervals following application in mice divided into five groups. The results show the effect of the formulations applied on microbial viability over time, as illustrated in Figure 5.

The results were normalized by setting the microbial viability at t = 0 to 100% and are presented as the percentage decrease in viability over time. In the control group (group 5), a gradual reduction in microbial viability was observed up to 30 min.

Cole et al. investigated the antimicrobial activity of native and manipulated nasal fluids against various bacterial strains. The antimicrobial activity of nasal secretions was evaluated at 0, 1, 3, and 24 h. In one sample, none were detected within 24 h. When this test was performed on another sample, a dramatic time-dependent decrease was observed, but little bacterial growth was observed at 24 h. These findings suggest that variations in nasal secretory composition may contribute to fluctuations in bacterial regrowth [31]. Therefore, the observed decrease in microbial viability in the control group may be attributed to the antimicrobial properties of nasal secretions. The antimicrobial effects of chitosan have been described in numerous studies [32–34]. When the impact of the chitosan ointment (group 4) on microbial viability was evaluated, a greater reduction was observed compared to the control group.

In terms of microbial viability, the thyme oil-containing ointment showed a greater antimicrobial effect than both the chitosan ointment and the control group. Compared with OLE ointment (group 2), it shows a higher antimicrobial effect at 30 min. Özoğul et al. investigated the antimicrobial activity of nanoemulsion containing thyme oil on fish spoilage bacteria and foodborne pathogens. Similar to our study, the main component of the thyme oil they used was carvacrol. Accordingly, the strong antibacterial activity was attributed to carvacrol, the major component of thyme oil [35]. Among the formulations tested, the OTC ointment (group 1) showed the greatest reduction in microbial viability over time. Compared to the control group and formulations containing individual components, the OTC formulation resulted in a more pronounced decrease in colony counts within the first 30 min following intranasal application. This effect may be attributed to the combined antibacterial contributions of chitosan and the encapsulated plant-derived bioactive compounds.

## 4. Conclusion

In the present study, a starch-based nasal ointment incorporating olive leaf extract and CMs encapsulating thyme oil was successfully developed and evaluated. The formulation was designed to form a physical barrier on the nasal mucosa while providing local antimicrobial activity. The physicochemical characterization demonstrated that the formulation selected was compatible with nasal application in terms of pH, viscosity, homogeneity, and physical appearance. In vivo evaluation further showed that the ointment containing olive leaf extract and thyme oil-encapsulated CMs led to a pronounced reduction in microbial viability shortly after intranasal application. These findings indicate that the combination of a barrier-forming nasal ointment base with chitosan-based microparticles carrying plant-derived compounds may offer a promising approach for limiting microbial presence in the nasal cavity. The results support the potential of this formulation as a local nasal barrier system with antimicrobial properties and provide a basis for further studies focusing on long-term efficacy and safety.

## Figures and Tables

**Table 1:** Compositions of the ointment formulations.

Formulation name	Chitosan microparticles	Olive leaf extract	Thyme oil	Ointment base
OTC ointment	+	+	+	+
OLE ointment	+	+	−	+
Thyme ointment	+	−	+	+
Chitosan ointment	+	−	−	+
Control ointment	−	−	−	+

**Table 2:** pH and viscosity values of the ointment formulations containing different OTC microparticle concentrations.

OTC concentration in the ointment base	pH	Viscosity (cP)
Control	6.5–7	2000 cP
%0.25	6	2500 cP
%0.5	6	2333.3 cP
%1	5–5.5	2109 cP
%2	4	1433.3 cP
